# Anti-thyroglobulin Antibodies as a Possible Risk Factor for Graves' Disease After Radioiodine Treatment for Toxic Nodular Goiter: Case Report

**DOI:** 10.3389/fnume.2022.858062

**Published:** 2022-03-15

**Authors:** Nathalie Rouiller, Marie Nicod Lalonde, Gerasimos P. Sykiotis

**Affiliations:** ^1^Service of Endocrinology, Diabetology and Metabolism, Lausanne University Hospital and University of Lausanne, Lausanne, Switzerland; ^2^Service of Nuclear Medicine and Molecular Imaging, Lausanne University Hospital and University of Lausanne, Lausanne, Switzerland

**Keywords:** Graves' disease, toxic nodular goiter, radioiodine, thyroid autoantibodies, thyroglobulin antibodies (TgAb)

## Abstract

**Background:**

The manifestation of Graves' disease (GD) in patients treated with radioactive iodine (RAI) for hyperfunctioning thyroid nodules (RAI-induced GD or post-RAI GD) remains a long-standing challenge in radionuclide therapy. Known risk factors for post-RAI GD include preexisting subclinical hyperthyroidism, positive thyroid peroxidase autoantibodies (TPOAb), positive TSH receptor autoantibodies (TRAb) or otherwise undiagnosed GD. However, these risk factors are not present in all patients with post-RAI GD, and therefore it cannot always be predicted in a reliable manner if a given patient has a high risk for RAI-induced GD or not.

**Case Presentation:**

We describe the case of a 64 year-old woman known for hyperthyroidism due to toxic nodular goiter; she was treated initially with carbimazole, and then, due to recurrence, underwent RAI treatment. Three months later, symptomatic hyperthyroidism persisted. Diagnosis of new-onset GD was made based on typical ultrasound findings and newly-positive TRAb. Our patient had only positive thyroglobulin antibodies (TgAb) before RAI treatment, whereas TPOAb were negative.

**Conclusions:**

In the literature, TgAb have never been reported as a possible risk factor for RAI-induced GD. The present case suggests that the assessment for pre-existing autoimmunity in patients considering RAI for hyperfunctioning thyroid nodules should probably also include TgAb.

## Introduction

New-onset Graves' disease (GD) after radioiodine (RAI) treatment for toxic nodular goiter (RAI-induced GD or post-RAI GD) is a rare phenomenon that is reported in the literature yet not broadly known among clinicians. Some risk factors have been described, such as the presence of autoantibodies against the TSH receptor (TRAb) or thyroid peroxidase (TPOAb) before RAI. However, these risk factors are not present in all patients with post-RAI GD, and therefore it cannot always be predicted in a reliable manner if a given patient has a high risk for RAI-induced GD or not.

We report here a patient with RAI-induced GD who had neither TRAb nor TPOAb, but only thyroglobulin antibodies (TgAb). In the literature, the presence of TgAb has not been previously described as a possible risk factor for RAI-induced GD.

## Case Presentation

**Figure 1:**
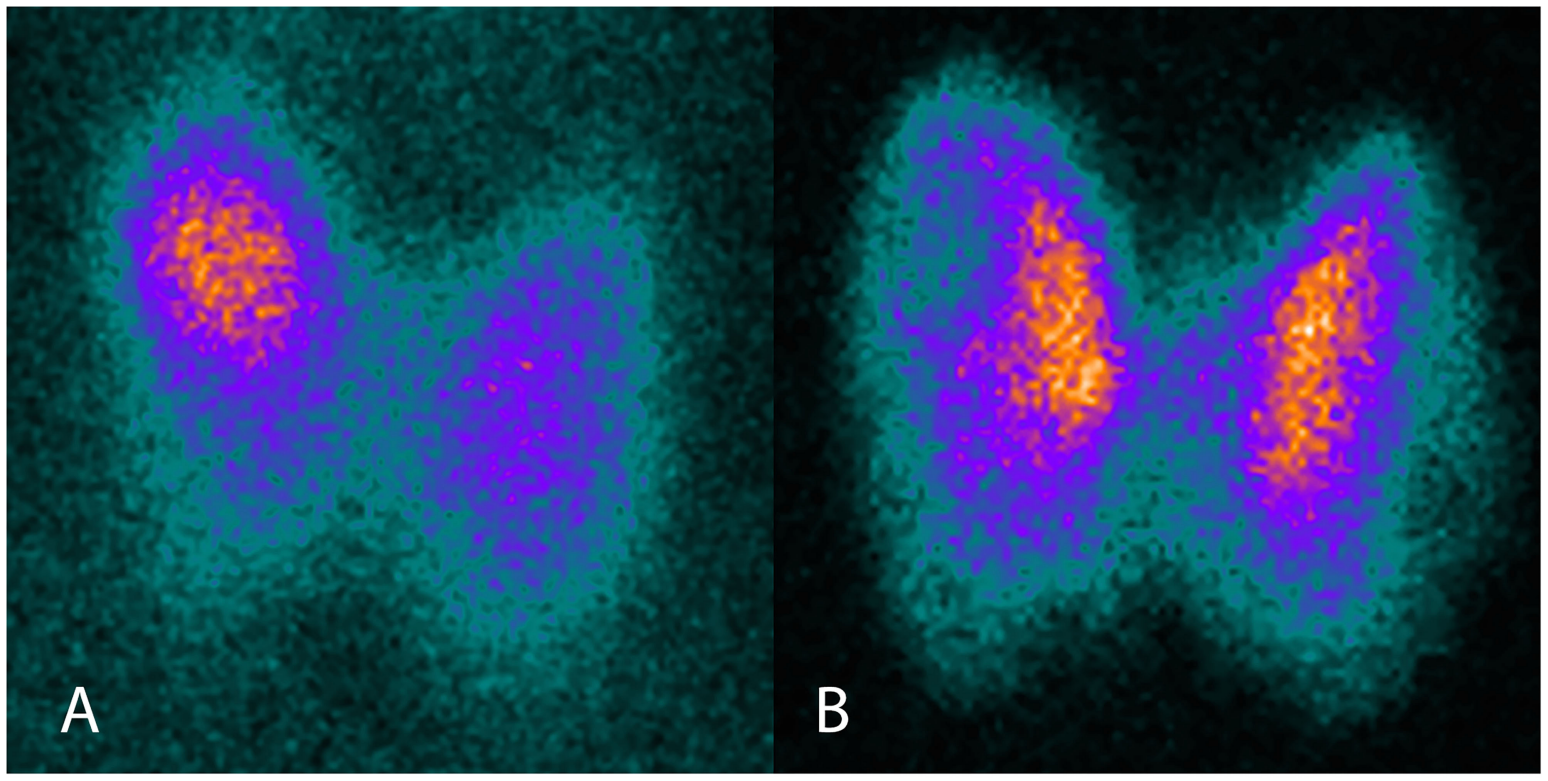
Thyroid scintigraphy before and after RAI therapy. **(A)** I-123 scintigraphy before RAI, showing focally increased uptake in the toxic adenoma. **(B)** Tc99m scintigraphy after RAI, showing diffusely increased uptake, except in the area of the previously treated toxic adenoma.

The patient was a 64 year-old woman known for toxic nodular goiter since 1997, documented by ultrasound and treated intermittently with carbimazole. Due to recurrence of hyperthyroidism in November 2016, ultrasound was repeated, showing a multinodular goiter, and thyroid scintigraphy was performed, showing a toxic nodule in the upper right thyroid lobe (Figure 1A). Uptake-based RAI treatment (367 MBq) was administered in January 2017, and levothyroxine substitution therapy was initiated soon thereafter. Three months later, the patient presented with symptomatic tachycardia and tremor and was referred to our hospital for evaluation and management. Thyroid hormone profiling revealed thyrotoxicosis that persisted after the suspension of levothyroxine substitution (Table 1). Thyroid palpation revealed diffuse goiter without palpable nodules. Ultrasound showed a multinodular goiter with marked heterogeneity in the extranodular thyroid parenchyma. The thyroid gland's vascularization was markedly increased, except in the area of the previously treated nodule. TRAb, which were negative before RAI treatment, were now positive. Thyroid scintigraphy showed diffusely increased uptake, except in the area of the previously treated nodule (Figure 1B). Due to carbimazole intolerance, total thyroidectomy was performed.

**Table 1:** Chronology of the patient's thyroid function tests and treatments.

**Date**	**TSH** **(0.27–4.2 mIU/L)**	**fT4** **(12–24 pmol/l)**	**fT3** **(3.1–6.8 pmol/l)**	**TRAb** **(<1 IU/l)**	**TPOAb** **(<5.6 IU/ml)**	**TgAb** **(<4.0 IU/ml)**	**Treatment**
05.10.16	0.075	14.9		<0.3	<1	5.5	
09.01.16							RAI, start LT4
13.02.17	0.007	28.9	7.8				Stop LT4
07.03.17	<0.005	31.0	17.0	4.7			
21.03.17	<0.005	59.8	26.5				Start carbimazole
02.05.17	<0.005	28.0	10.0	17.0			Carbimazole

*fT3, free T3; fT4, free T4; LT4, levothyroxine; TgAb, thyroglobulin antibodies; TPOAb, thyroid peroxidase antibodies; TRAb, TSH receptor antibodies; TSH, thyroid-stimulating hormone; RAI, radioactive iodine*.

When the patient's medical record was retrospectively examined for possible risk factors of RAI-induced GD, it was noted that TPOAb were negative before RAI, and only TgAb were slightly positive (Table 1).

## Discussion and Conclusions

After RAI treatment for toxic adenomas or toxic multinodular goiter, the majority of patients present euthyroidism or hypothyroidism (1). Persistent thyrotoxicosis following RAI treatment may occur in various situations: (i) In patients substituted with levothyroxine, overtreatment must be ruled out. In our patient, no improvement was noticed after stopping levothyroxine. (ii) Subacute thyroiditis should be excluded based on clinical and ultrasonographic findings. Our patient had no cervical pain and the thyroid's vascularization was increased, rather than decreased or absent, arguing against subacute thyroiditis. (iii) Insufficient RAI treatment is another theoretical possibility. In our patient, scintigraphy, in correlation with ultrasound, confirmed the non-functional state of the previously toxic nodule and the absence of new autonomous nodules. (iv) RAI-induced GD should be distinguished from the Marine-Lenhart syndrome, in which toxic nodules coexist with GD; conversely, an unusual case of Marine-Lenhart syndrome has been reported, in which an autonomous thyroid nodule developed after RAI treatment for GD (2). In our patient, there were no clinical signs (i.e., orbitopathy and dermatopathy), biochemical markers (i.e., TRAb) or imaging findings (i.e., diffuse/patchy autonomy on scintigraphy) of GD before RAI treatment. (v) Thyrotoxicosis may be observed early after RAI treatment, secondary to RAI-induced inflammation (actinic thyroiditis) that results in release of thyroid hormones (3). This phenomenon is typically observed during the first days or weeks following the treatment. Our patient presented thyrotoxicosis 3 months after RAI treatment, which is not typical of actinic thyroiditis but is quite typical of RAI-induced GD (4). (vi) Finally, as in most cases of thyrotoxicosis, TRAb must be measured. In our patient, the constellation of a typical ultrasound pattern, newly-positive TRAb and diffuse autonomy on scintigraphy confirmed the diagnosis of new-onset GD that occurred 3 months after RAI treatment, thus classifying this patient as a case of RAI-induced GD.

RAI-induced GD is uncommon, with an incidence that is variable among studies and ranges from 0 to 5.4% (4–6). The pathogenetic mechanisms of this phenomenon are not yet clearly established, but some pathophysiological explanations have been proposed. An exacerbation of a pre-existing subclinical or undiagnosed GD is suggested as a potential cause of new-onset GD, which, strictly speaking, should be distinguished from new-onset RAI-induced GD. It is important to keep in mind that TRAb may remain below threshold in mild forms of GD or when using less sensitive immunoassays (5, 7, 8). Thyroid autoimmunity is exacerbated after RAI treatment due to release of thyroid antigens as a result of follicular cell destruction (5, 7). This exacerbation of autoimmunity has also been observed after external radiation for non-thyroidal illness, subacute thyroiditis, surgical manipulation of the thyroid during parathyroidectomy, or percutaneous ethanol injection treatment (8, 9). A diffuse/patchy uptake pattern on scintigraphy before RAI treatment may be a risk factor to develop new-onset GD (5, 10, 11), ostensibly due to the more extensive cellular destruction expected in such cases. The presence of TPOAb or TRAb before RAI treatment attests to the presence of thyroid autoimmunity and represents a risk factor to develop RAI-induced GD. Indeed, the presence of TPOAb before RAI treatment increases this risk ~10-fold (5, 6). Interestingly, before RAI treatment, our patient had neither TRAb nor TPOAb but only TgAb. TgAb have not been described or assessed as a possible risk factor to develop RAI-induced GD, neither in case reports nor in cohort studies, and a recent systematic review makes no mention of them either (4). The estimated prevalence of positive TPOAb in Hashimoto's disease is 95 vs. 80% for TgAb (12); therefore, TgAb are considered less sensitive for the detection of autoimmune thyroid disease and are not measured systematically.

In conclusion, while RAI treatment is an excellent therapeutic option for toxic adenoma and toxic multinodular goiter, the risk of RAI-induced GD is not negligible. Assessment of this risk in individual patients should comprise evaluation of clinical, biochemical, ultrasonographic and scintigraphic markers of thyroidal autoimmunity. The present case report suggests that the biochemical profiling should include the measurement of TgAb, and that their positivity may possibly increase the risk of RAI-induced GD even in the absence of other, better established risk factors.

## Data Availability Statement

The original contributions presented in the study are included in the article/supplementary material, further inquiries can be directed to the corresponding author.

## Ethics Statement

Written informed consent was obtained from the relevant individual for the publication of any potentially identifiable images or data included in this article.

## Author Contributions

NR, MN, and GS analyzed and interpreted the patient data. NR and GS drafted the manuscript. All authors read, edited, and approved the final manuscript.

## Funding

GS was supported by a 2016 Leenaards Foundation Fellowship for Academic Promotion in Clinical Medicine. The funder provided salary support to secure protected research time for GS. The funder had no direct involvement in the preparation of the present article.

## Conflict of Interest

The authors declare that the research was conducted in the absence of any commercial or financial relationships that could be construed as a potential conflict of interest.

## Publisher's Note

All claims expressed in this article are solely those of the authors and do not necessarily represent those of their affiliated organizations, or those of the publisher, the editors and the reviewers. Any product that may be evaluated in this article, or claim that may be made by its manufacturer, is not guaranteed or endorsed by the publisher.

## Abbreviations

GD: Graves' disease
fT3: free T3
fT4: free T4
LT4: levothyroxine
RAI: radioactive iodine
TgAb: thyroglobulin antibodies
TPOAb: thyroid peroxidase antibodies
TRAb: TSH receptor antibodies
TSH: thyroid-stimulating hormone.
